# Magnetically retrievable Ce-doped Fe_3_O_4_ nanoparticles as scaffolds for the removal of azo dyes†

**DOI:** 10.1039/c9ra03252e

**Published:** 2019-07-26

**Authors:** Aashima, Shivani Uppal, Arushi Arora, Sanjeev Gautam, Suman Singh, R. J. Choudhary, S. K. Mehta

**Affiliations:** Department of Chemistry, Centre of Advanced Studies in Chemistry, Panjab University Chandigarh 160014 India skmehta@pu.ac.in; Dr S. S. Bhatnagar University Institute of Chemical Engineering and Technology (SSB UICET), Panjab University Chandigarh 160014 India; CSIR – Central Scientific Instruments Organization Sector-30 Chandigarh 160030 India; UGC-DAE Consortium for Scientific Research University Campus, Khandwa Road Indore – 452 017 India

## Abstract

Considering the significant impact of magnetically retrievable nanostructures, herein, Fe_3_O_4_ and Ce-doped Fe_3_O_4_ nanoparticles were employed as scaffolds for the removal of the Reactive Black 5 (RB5) azo dye. We synthesized the Ce-doped Fe_3_O_4_ nanoparticles *via* hydrothermal treatment at 120 °C for 10 h with varying cerium concentrations (1.5–3.5%) and characterized them using basic techniques such as FTIR and UV-visible spectroscopy, and XRD analysis. The retention of their magnetic behaviors even after cerium amalgamation was demonstrated and confirmed by the VSM results. FESEM and EDX were used for the morphological and purity analysis of the synthesized nanoabsorbents. XPS was carried out to determine the electronic configuration of the synthesized samples. The porosity of the magnetic nanoparticles was investigated by BET analysis, and subsequently, the most porous sample was further used in the adsorption studies for the cleanup of RB5 from wastewater. The dye adsorption studies were probed *via* UV-visible spectroscopy, which indicated the removal efficiency of 87%. The prepared Ce-doped Fe_3_O_4_ nanoabsorbent showed the high adsorption capacity of 84.58 mg g^−1^ towards RB5 in 40 min. This is attributed to the electrostatic interactions between the nanoabsorbent and the dye molecules and high porosity of the prepared sample. The adsorption mechanism was also analyzed. The kinetic data well-fitted the pseudo-first-order model, and the adsorption capability at different equilibrium concentrations of the dye solution indicated monolayer formation and chemisorption phenomena. Furthermore, the magnetic absorbent could be rapidly separated from the wastewater using an external magnetic field after adsorption.

## Abstract 2

Considering the significant impact of magnetically retrievable nanostructures, herein, Ce-doped Fe_3_O_4_ nanoparticles were employed as scaffolds for the removal of the Reactive Black 5 (RB5), an azo dye.

## Graphical abstract

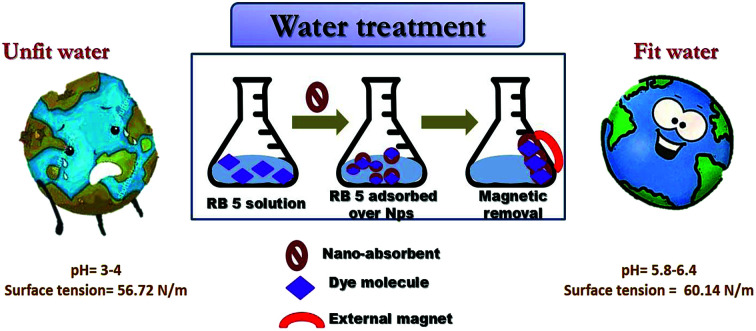

## 1. Introduction

Synthetic dyes have provided us with the advantage of achieving fast and brighter colors. Dyestuffs require a large amount of water for dyeing. About 80% of dyestuffs stay on the substrate, whereas the rest just go down the drain.^1^ With the development of the printing and dyeing industry, wastewater pollution is posing a threat with dyes as typical organic pollutants. The toxic nature of dyes has become a cause of serious concern to environmentalists. The use of synthetic dyes has an adverse effect on all life forms such as humans, animals or plants, and the grave outcomes necessitate their effective disposal; azo dyes bear an azo (–N=N) functional group together with complex chemical moieties comprising phenyl (C_6_H_5_CH_2_), naphthyl (C_10_H_7_CH_2_) or phenylamine (C_6_H_5_–NH_2_) groups;^2^ these moieties are highly complex for treatment *via* traditional biological methods. Thus, various chemical and physical treatment methods, such as coagulation–flocculation,^3^ advanced oxidation,^4^ membrane filtration,^5^ adsorption on activated carbon,^6^ and electrochemical^7^ and catalysis approaches,^8^ have long been established for the decolorization of textile wastewater. However, most of these methods suffer from high cost of experimentation, use of sophisticated instruments and manpower and inability to meet the permissible disposal levels. Most importantly, they have unsatisfactory recyclability and inefficient regeneration, which restrict their application in an industrial setup.

Adsorption has been commonly used as an effective technique for the removal of dyes. Activated carbon,^9^ rice husk,^10^ metal hydroxide sludge,^11^ zeolites^12^ and other compounds derived from natural sources have been used as adsorbents. Moreover, the adsorption process is preferred over the degradation of azo linkages since various reactive species, *i.e.* hydroxyl and hydrogen radicals, are involved in the latter process that lead to various degradation products (*e.g.* arylamines), which are potentially more toxic than the dyestuff itself.^13^

Magnetic nanoparticle (Np) adsorption has unfolded as an auxiliary method to usual adsorption for the treatment of effluent wastewater;^14^ hence, magnetic nanoadsorbents can serve as rapid dye adsorbing agents for the adsorption of dyes from wastewater effluent due to the presence of hydroxyl groups on their surfaces and consequently can be easily retrieved from the medium using a magnetic field post implementation. Some studies have been reported on the adsorption of pollutants on magnetite-based composites.^15–17^ Magnetic lignin-based adsorbents have been synthesized, and their adsorption capacity has been found to be more than 90% for azo dyes.^18^ Elwakeel *et al.*^19^ used magnetic chitosan resins to remove Reactive Black 5 (RB5) from aqueous solutions and were able to efficiently elute the adsorbate back. Chatterjee *et al.*^20^ used modified zero-valent iron with various surfactants, such as cetyltrimethylammonium bromide and sodium dodecyl sulfate, for the removal of RB5 from wastewater. Pure magnetite (Fe_3_O_4_) exhibits excellent magnetic properties; however, its adsorption efficiency towards pollutants is low.^21^ Thus, doping of other metals/metal ions, such as zinc and cobalt, into the Fe_3_O_4_ matrix increases the availability of its surface sites; this accordingly improves the adsorption capacity of Fe_3_O_4_.^22,23^ Furthermore, Su *et al.*^24^ reported the relationship between manganese doping and adsorption properties. The maximum adsorption capacity was found to be 84.54 mg g^−1^ in the case of doped hematite as compared to the case of undoped hematite (33.02 mg g^−1^). Vanadium-doped Fe_3_O_4_ promoted the adsorption of methylene blue with a high degree of decolouration. The improved adsorption efficiency was due to the increase in superficial hydroxyl groups.^25^

In the present study, we fabricated Fe_3_O_4_ and Fe_3_O_4_ doped with cerium (Ce), Ce–Fe_3_O_4_, *via* a hydrothermal treatment. Ce exhibits a facile transition between the +3 and +4 oxidation states, and the differences in the oxidation states of the host and dopant metal leads to the generation of octahedral (O_v_) defects, which act as charge compensating vacancies and tend to influence various properties. The synthesized samples were well characterized by XRD, UV-vis spectroscopy, N_2_ adsorption/desorption, FTIR spectroscopy, FE-SEM and XPS. The concentration of cerium as a dopant was varied, and XRD was conducted to confirm the doping. The magnetic behavior was analyzed using VSM studies. Furthermore, the effect of cerium concentration on various properties, such as magnetic and surface area properties, was investigated. Reactive Black 5 (RB5), an anionic dye that belongs to the category of azo-reactive dyes and is extensively used in textile industries for dyeing, has been selected as a model dye. This study highlights the potential application of Ce-doped Fe_3_O_4_ Nps as efficient RB5 adsorbents and their magnetic retrieval with the retention of their properties, which widens their scope for application in environmental remediation processes.

## 2. Experimental

### 2.1. Materials

Iron(iii) chloride hexahydrate (FeCl_3_·6H_2_O, 97%), iron(ii) sulphate hexahydrate (FeSO_4_·6H_2_O, ≥99%) and Reactive Black 5 were purchased from Sigma-Aldrich. Aqueous ammonia (25%) was purchased from Merck. Cerium(iii) nitrate hexahydrate (Ce(NO_3_)_3_·6H_2_O, ≥99%) was obtained from HPLC Pvt. Ltd. All chemicals were of analytical grade and used without further purification. Deionised water (DW) was used for the synthesis and sample preparation.

### 2.2. Synthesis of Fe_3_O_4_ and Ce–Fe_3_O_4_ Nps through the hydrothermal treatment

**Scheme 1:**
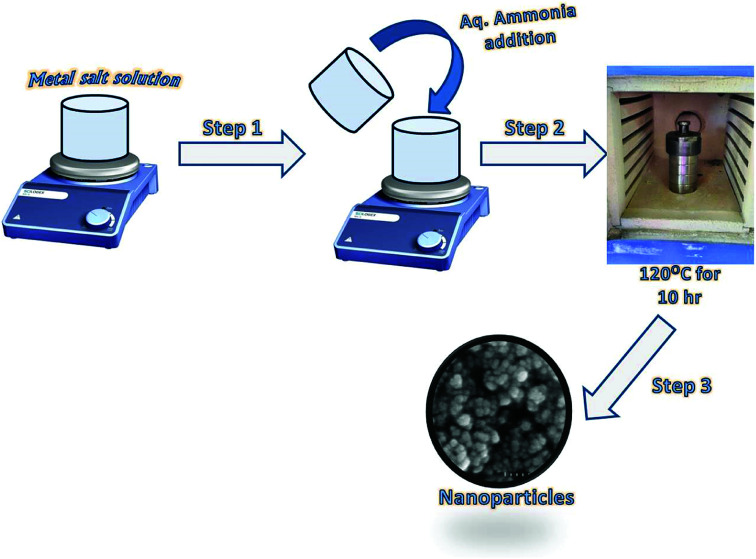
Schematic for the fabrication of Fe_3_O_4_ and Ce–Fe_3_O_4_ Nps.

The synthesis of Fe_3_O_4_ and Ce–Fe_3_O_4_ Nps was performed by a hydrothermal method (Scheme 1). In a typical reaction process, FeCl_3_·6H_2_O (0.270 g) and 0.139 g of FeSO_4_·6H_2_O solutions were dissolved in 10 mL distilled water. The mixture was then stirred for 5 min, followed by the dropwise addition of 5 mL of NH_3_ solution (25%). To this mixture, Ce(NO_3_)_3_·6H_2_O at different concentrations, obtained by dissolving different amounts of it ranging from 1.5 to 3.5 wt% in 10 mL distilled water, was added followed by stirring for 30 min at room temperature. The obtained solution was then transferred to a Teflon-lined stainless-steel autoclave and placed in a furnace at 120 °C for 10 h. The autoclave was allowed to naturally cool down to room temperature after the hydrothermal treatment. The precipitate was recovered, washed several times with distilled water and finally with ethanol to remove the impurities and dried in an oven at 60 °C.

### 2.3. Characterization techniques

X-ray diffraction studies were performed using the PANalytical X'pert PRO XRD at the scanning speed of 8° min^−1^ with Cu-Kα radiation (1.5418 Å) in the 2*θ* range of 10–80°. The FTIR spectra were obtained using a thermally controlled diode laser in the transmittance mode in the spectral region of 4000–500 cm^−1^ using Thermo Scientific, Nicolet iS50 FTIR. The UV-visible absorption spectra were obtained using the JASCO V 530 spectrophotometer (4-21, Sennin-cho 2-chome, Hachioji, Tokyo 193-0835, Japan model). The spectral range of 190–450 nm was covered with the precision of ±0.2 nm using quartz cuvettes having 1 cm path length. The Lake Shore Vibrating Sample Magnetometer (VSM) was used to measure the magnetization as a function of the applied field and temperature. Malvern Zeta Nano S90 (Malvern Instruments, Malvern, UK), which calculates intensity weighted mean particle size (*Z*-average, nm ± S.D.), has been used to obtain the particle size distribution of the sample. The surface morphology of the sample was analyzed using the Hitachi-SU8010 field-emission electron microscope (FE-SEM), operating at the voltage of 15 kV. Electron micrographs were obtained by transmission electron microscopy (TEM-TECNAI 200 kV). Energy dispersive X-ray spectroscopy analysis was carried out using Bruker-Xflash. The BET surface areas of the samples were analyzed by nitrogen adsorption–desorption using a N_2_ adsorption analyzer, BELSORP. After degassing all the samples at 423 K for 15 hours, the BET surface area was determined using the multipoint BET method. The Barrett–Joyner–Halenda (BJH) method was used to analyse the pore-size distribution using the adsorption branch of the isotherm. X-ray photoelectron spectroscopy (XPS) was performed using the Omicron energy analyzer (EA-125) with an Al Kα (1486.6 eV) X-ray source. The sample was sputtered with Ar^+^ for 5 min before the spectra were obtained. The background vacuum in the analyzer chamber was of the order of 10^−10^ Torr during the XPS measurement.

### 2.4. Dye adsorption experiments

All experiments were performed at room temperature under light. For adsorption studies, at first, 10 mg of the synthesized sample was added to 50 ppm of dye solution, *i.e.* RB5, to confirm the adsorption capability of the synthesized nanomaterials. The dye concentration was 30 ppm for all the dyes employed herein. The pH value was maintained between 3 and 4. The color of the RB5 solution faded with time, confirming adsorption. Initially, various optimization parameters were investigated by analysing the effect of contact time, pH and dosage on adsorption. For adsorption capacity calculations, the UV-visible absorbance was measured every 5 min by pipetting out 1 mL solution and then filtering it. The equilibrium adsorption amount (*Q*_e_) of the 3.5% Ce–Fe_3_O_4_ Nps for the RB5 dye was calculated using the following eqn (1):

1 *Q*_e_ = *v*(*C*_o_ − *C*_e_)/*m*

where *C*_o_ and *C*_e_ are the initial and equilibrium concentrations (mg L^−1^) of the dye solution, respectively, *v* corresponds to the volume of the dye solution, and *m* refers to the weight of the Nps.

## 3. Results and discussion

### 3.1. Physical characterization of the synthesized samples

#### 3.1.1. X-ray diffraction

**Fig. 1:**
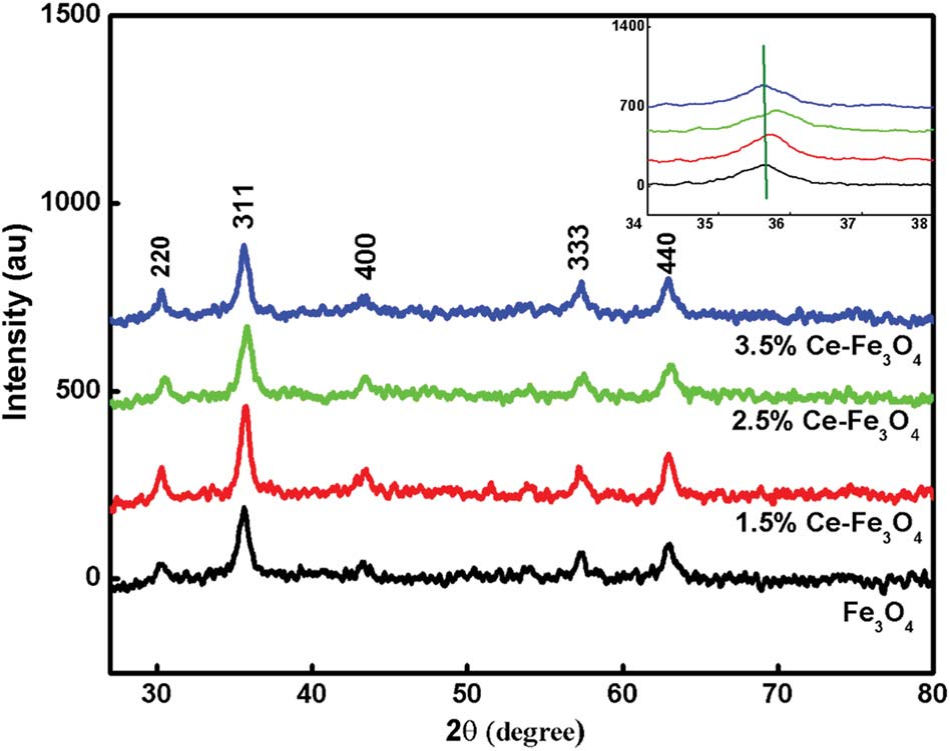
XRD pattern of the pure Fe_3_O_4_ and doped synthesized samples.

The XRD patterns of the Fe_3_O_4_ and Ce–Fe_3_O_4_ samples are shown in Fig. 1. The X-ray diffractograms of the polycrystalline samples reveal the generation of an unambiguous single-phase inverse spinel structure. The XRD pattern of the undoped Fe_3_O_4_ sample exhibits diffraction peaks at about 30.39°, 35.66°, 43.15°, 53.96°, 57.30° and 62.94°, corresponding to the (220), (311), (400), (422), (511) and (440) planes; this is agreement with literature (JCPDF card, file no. 74-0748); all the observed peaks have been indexed to pure Fe_3_O_4_, and no diffraction peaks corresponding to oxidized cerium species are found; the XRD peaks provide the requisite information about the location of the dopant in the crystal lattice. The ion distribution between the tetrahedral and octahedral sites is determined by the relative size and charge on/of cations and the size of the interstices.^26,27^ In the Ce–Fe_3_O_4_ system, the Fe^2+^ ions show high crystal field stabilization energy (CFSE), whereas the Fe^3+^ and Ce^4+^ ions have zero CFSE at both the octahedral and the tetrahedral sites. Therefore, considering the CFSE and ionic radii, it is reasonable that Ce^4+^ enters the octahedral sites. The substitution can change the 2*θ* values, as shown in the inset of Fig. 1; this is indicative of cation substitution since upon substitution, the crystallite size changes, resulting in a change in scattering.^35^

With an increase in the dopant concentration, an increase in crystallinity was observed; this was indirect evidence of the decrease in the crystallite size of the synthesized samples. The crystallite size was calculated using the Debye–Scherrer equation as follows:2
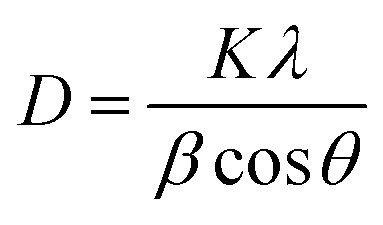
where the parameter *D* is the crystallite size, *λ* is the wavelength of X-rays used (0.154 nm), *θ* is the Bragg's diffraction angle and *β* is the full-width-at-half maxima (FWHM). The average crystallite size was calculated and is presented in Table 1. The crystallite size was found to increase; this could be ascribed to the slight difference in the ionic radii of both cations, which made the substitution possible. If the ionic radii of the two ions differ by less than 14%, crystal chemistry permits the miscibility and substitution of ions.^28^ Therefore, we can conclude that substitution of ions in the crystal lattice occurs without the formation of any other phase.

**Table 1:** Crystallite size calculated from the XRD pattern

System	Crystallite size (nm)
Fe_3_O_4_	25.33
1.5% Ce–Fe_3_O_4_	24.88
2.5% Ce–Fe_3_O_4_	23.22
3.5% Ce–Fe_3_O_4_	23.09

### 3.2. Fourier transform infrared spectroscopy (FTIR)

**Fig. 2:**
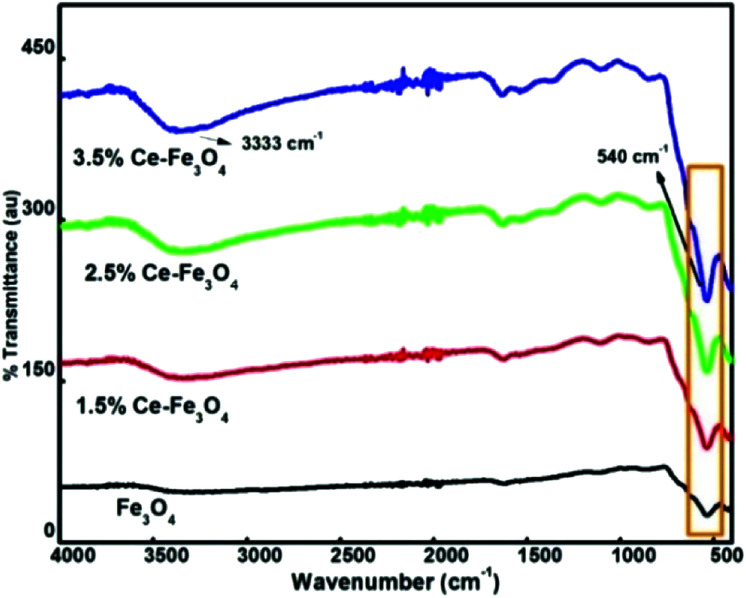
FTIR spectra of pure Fe_3_O_4_ and doped Fe_3_O_4_.

FTIR spectra provide details about the molecular structure and its environment since it is sensitive to various chemical bonds and functional groups present in a molecule. The FTIR spectra of Fe_3_O_4_ and Ce–Fe_3_O_4_ Nps are depicted in Fig. 2. Fe_3_O_4_ exhibits^29^ only two bands between 400 and 800 cm^−1^. The peak at 1614 cm^−1^ and the broad band between 3200 and 3500 cm^−1^ are attributed to the stretching and bending vibrations of the hydroxyl groups,^30,31^ which indicate the existence of water molecules adsorbed on the surface of Fe_3_O_4_ and Ce–Fe_3_O_4_, respectively. This is mainly due to chemical co-precipitation, where particles are naturally covered with hydroxyl groups. The characteristic peak at 540 cm^−1^ with a shoulder at 625 cm^−1^ corresponds to the intrinsic stretching vibrations of metal–oxygen at the tetrahedral site.^32^

In addition, the low frequency peak observed at around 445 cm^−1^ is assigned to the metal–oxygen stretching vibration at the octahedral site. The band disturbances below 1500 cm^−1^ are due to the lowering of the local symmetry as a result of distortion due to the presence of different cations at different sites^33^ (Fig. 2). Moreover, the bands near 540 cm^−1^ become less intense and broader because of the disturbance of the local symmetry *via* the introduction of Ce^4+^ into the lattice. The absorption band at 540 cm^−1^ gradually shifted; this was attributed to the revamped bonding force between the cations and oxygen anions because of the presence of cerium ions.

### 3.3. UV-visible spectra

The UV-vis analysis helps to understand the optical behaviour of materials. Thus, in this study, the absorbance of Fe_3_O_4_ and Ce–Fe_3_O_4_ Nps was monitored at ambient temperature (Fig. S1†). Magnetite exhibits thermally induced electron delocalization between adjacent Fe^2+^ and Fe^3+^ ions, and its electronic transitions are assigned to intervalence charge transfer (IVCT) transitions in the visible and near-IR region.^34^ The optical properties were enhanced by the introduction of Ce into the lattice of Fe_3_O_4_, as can be observed by the intensity enhancement. This can be accredited to the fact that new defects are introduced after the substitution of Ce cations into the lattice unit.

### 3.4. Vibrating sample magnetometer (VSM)

**Fig. 3:**
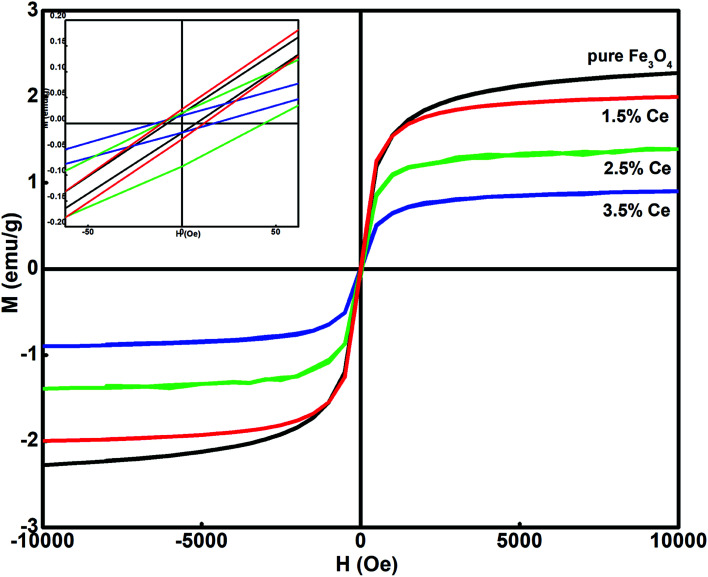
Hysteresis loop at room temperature for the synthesized samples.

Fig. 3 shows the dependence of magnetization on the applied field (*M*–*H*) for all the synthesized samples at room temperature. All the samples displayed ferromagnetic behaviour. The hysteresis loop was used to calculate the magnetism saturation (*M*_s_) and coercivity (*H*_c_), as listed in Table 2. The undoped Fe_3_O_4_ sample displayed better *M*_s_ than the doped samples. The Ce-doped Fe_3_O_4_ sample exhibited decreased magnetism due to the presence of surface defects, which caused a decrease in the exchange coupling between neighbouring iron ions on the surface.^35^ This occurs due to the lack of an oxygen super exchange mechanism. Surface spin disorder is caused by the broken exchange bonds on the surface of the particles, which influences magnetism.^36^ It has been reported that spin canting reduces after rare earth doping and thus increases the magnetization.^37^ The decrease in *M*_s_ with an increase in the doping level is attributed to the pronounced effect of the f-electrons in cerium.^38^ As f-electrons are deep- seated, they produce an opposite magnetic field, which cancels the effect of the overall magnetic field produced by a domain, and hence, the saturation magnetization becomes smaller. Another reason may be that with a decrease in particle size, an increase in surface to volume ratio occurs, *i.e.* the dead surface layers contribute more to the magnetism than the internal layers. Therefore, due to the dominant role of the dead surface layers in the particle gross magnetic property, smaller particle size results in a decrease in saturation magnetization; all the synthesized samples exhibit magnetic behavior; this makes their separation easy after treatment.

**Table 2:** Effect of cerium doping on the synthesized samples

System	Saturation magnetization, *M*_s_ (emu g^−1^)	Magnetic moment	Coercivity, *H*_c_ (Oe)
Fe_3_O_4_	11.970	0.507	7.004
1.5% Ce–Fe_3_O_4_	7.298	0.309	8.083
2.5% Ce–Fe_3_O_4_	4.326	0.183	10.776
3.5% Ce–Fe_3_O_4_	2.959	0.125	15.567

### 3.5. Morphological analysis: field-emission scanning electron microscopy (FE-SEM), transmission electron microscopy and energy dispersive X-ray (EDX) analysis

**Fig. 4:**
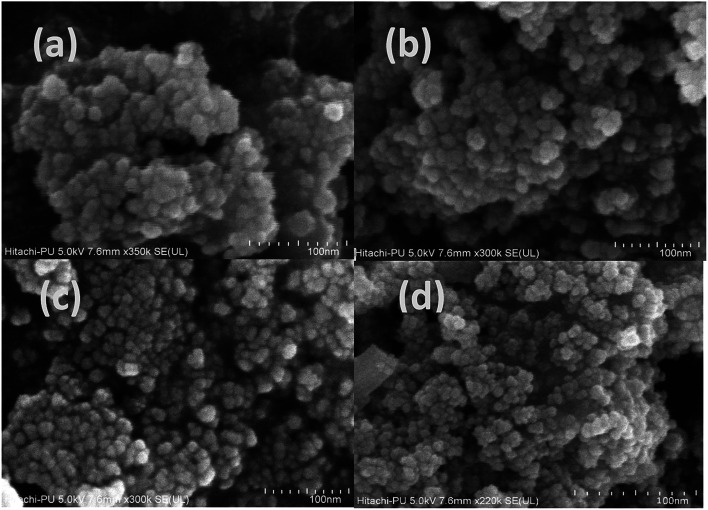
FE-SEM analysis of the (a) undoped and (b) 1.5%, (c) 2.5%, and (d) 3.5% cerium-doped samples.

The morphological characterization of the synthesized samples was carried out by obtaining their FE-SEM (Fig. 4) and TEM images (Fig. S2†). The results of both analysis reveal that the synthesized samples have a spherical, loose, aggregated structure with the average size of 24 ± 2 nm in the case of the undoped sample and 29 ± 0.9 nm for the 3.5% doped sample (Fig. S2†). As the dopant concentration increased, although the morphology remained unchanged, the particle size increased. This is also indicative of a slight change in aggregation. The results obtained *via* FESEM and TEM support those obtained *via* the XRD analysis; this reveals that an increase in size occurs with an increase in dopant concentration.

The EDX spectrum of Fe_3_O_4_ shows the presence of Fe and O and that of Ce–Fe_3_O_4_ shows the presence of Ce along with Fe and O (Fig. S3a†).

### 3.6. Brunauer–Emmett–Teller (BET) isotherm analysis

**Fig. 5:**
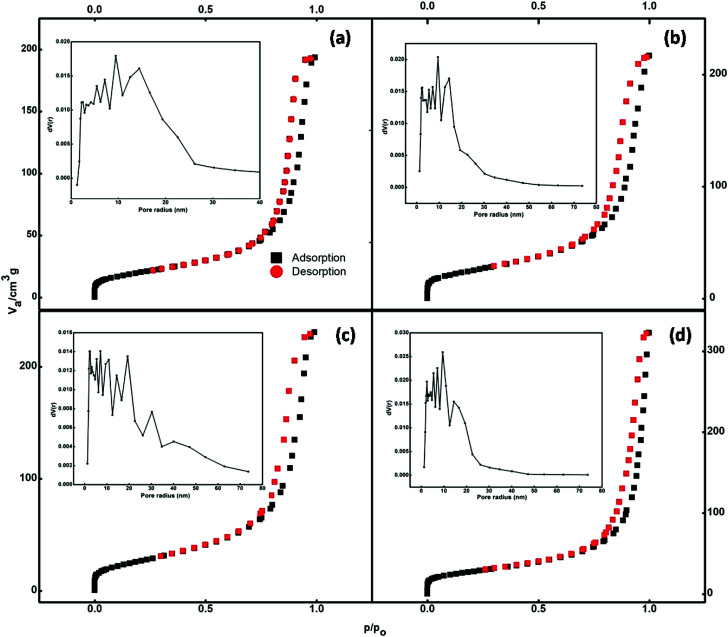
BET curves of (a) undoped and (b) 1.5%, (c) 2.5%, and (d) 3.5% cerium-doped samples.

The nitrogen adsorption–desorption isotherms of the prepared samples are illustrated in Fig. 5. According to the IUPAC classification, the isotherms shown in Fig. 5 can be classified as type IV. Type IV isotherms are particularly observed in the case of mesoporous adsorbents, which have a pore size between 2 and 50 nm. The adsorbent adsorptive interactions along with the interactions between molecules determine the adsorption behaviour in the mesopores in the condensed state. In the pore condensation phenomenon, gas condenses into a liquid-like phase in a pore at the pressure *p*, less than the saturation pressure *p*_0_ of the bulk liquid.^39^ Thus, final saturation plateaus of variable length are a typical feature of type IV isotherms.

Hysteresis is accompanied by the phenomenon of capillary condensation in the case of type IV isotherms.^40^ The mean pore diameter of the synthesized samples ranged between 14 and 19 nm (Table S1†). The void spaces between nanoparticles that constitute the nanospheres create these pores.

The pore size distributions of the catalysts were calculated by the Barrett–Joyner–Halenda (BJH) curve (inset of Fig. 5). It is evident that the adsorption capacities of the pure Fe_3_O_4_ and Ce-doped Fe_3_O_4_ samples increase with equilibrium pressure; this may be attributed to the condensation of N_2_ molecules in mesopores at high pressures. Doping with Ce increased the specific surface area (Table S1†) available for adsorption from 71.6 to 97.6 cm^3^ g^−1^, implying that 3.5% Ce–Fe_3_O_4_ could facilitate the diffusion and adsorption of the target pollutant; this would be helpful for the elevation of adsorption capacity. The BET specific surface area, which is an important property for adsorption studies, is high; this may be due to porosity resulting from the aggregation of magnetic Nps. Therefore, the system with maximum surface area (3.5% Ce–Fe_3_O_4_) is favorable for adsorption applications and has been chosen for further studies, *i.e.* adsorption of RB5 from wastewater.

### 3.7. X-ray photoelectron spectroscopy (XPS) analysis

**Fig. 6:**
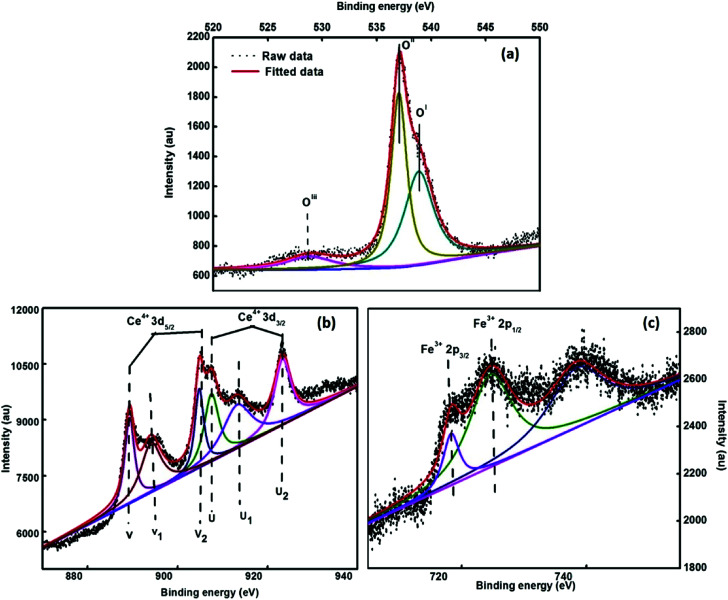
XPS spectra of the 3.5% cerium-doped sample (a) O 1s, (b) Fe 2p and (c) Ce 3d.

To evaluate the elemental composition, chemical environment and oxidation states of the various elements present in the synthesized system, the XPS spectra were obtained. Fig. 6 depicts the deconvoluted XPS spectra of 3.5% Ce–Fe_3_O_4_, *i.e.* Ce 3d, O 1s and Fe 2p states of the elements present in the examined sample. The peak centered around 537.3 eV (O^II^) is attributed to the O^2−^ ions attached to the Fe atoms (Fig. 6a). The intense peak at 538.1 eV (O^I^) results from the configuration and photo ionization of the 1s core level in the case of molecular oxygen.^41^

The peak around 528.06 eV (O^III^) is due to oxygen present in the cerium oxide lattice,^42^ which indicates the presence of cerium oxide as an impurity. The ratio of the oxygen peaks (O^II^/O^III^) in the iron and cerium lattice depicts that the amount of cerium oxide is very less. The peaks observed at 883.4 eV and 901.6 eV (Fig. 6b) are due to the spin–orbit coupling of the 3d_5/2_ and 3d_3/2_ levels, respectively. The peaks located at 883.4 eV, 889.9 eV, 899 eV, 901.6 eV, 908.4 eV and 917.5 eV are ascribed to the 3d^10^4f^1^ electronic state of Ce^4+^, and the peaks positioned at 885.06 eV and 902.8 eV are attributed to the 3d^10^4f^1^ states of the Ce^3+^ ion.^43^ The Fe 2p core level XPS spectrum reveals the peaks of Fe 2p_3/2_ and Fe 2p_1/2_ at 718.3 eV and 724.5 eV, which are attributed to the Fe^3+^ state of Fe^44^ (Fig. 6c). Fe 2p_3/2_ does not have a satellite peak;^44^ this confirms the formation of Fe_3_O_4_ in the present study.

### 3.8. Adsorption studies of 3.5% Ce–Fe_3_O_4_

**Fig. 7:**
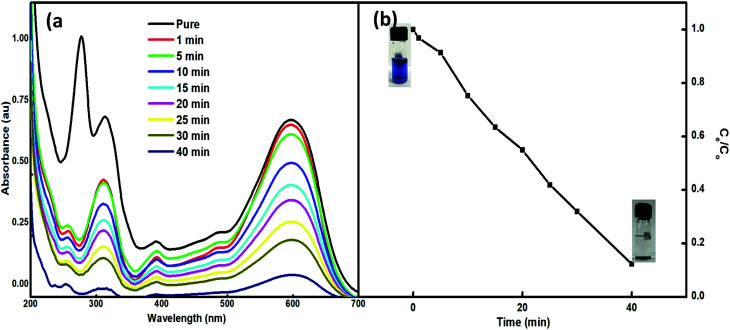
(a) UV-visible spectra of RB5 in the presence of 5 mg of Nps within time interval of 1–40 min and (b) adsorption profile of RB5 with time.

The feasibility of the synthesized nanoparticles as proficient nanoabsorbents for the removal of Reactive Black 5 (RB5), a textile azo dye, was explored. The strong adsorption capacity of Nps is ascribed to the ionic interactions between the dye and the nano-absorbent. The higher charge and porosity of the nanoabsorbent led to high removal efficiency. The adsorption peak at 600 nm in the visible spectrum of an aqueous solution of RB5 was obtained using 5 mg of Nps and found to decrease with time (1–90 min) (Fig. 7). For this, the absorbance of RB5 was monitored in the presence of the nanoadsorbent. The higher charge and porosity of the presented nanoadsorbent facilitated the removal of the dye through strong ionic interactions between the dye and Ce–Fe_3_O_4_. Fig. 7 shows the absorbance spectra in the presence and absence of the nanoadsorbent.

#### 3.8.1. Effect of operational parameters on the removal of dye

To optimize the adsorption ability of the nanoadsorbent for RB5, the effect of various operational parameters, such as pH, dosage, dye concentration and contact time, on the removal of the dye was examined at room temperature.

##### 3.8.1.1. Effect of pH

**Fig. 8:**
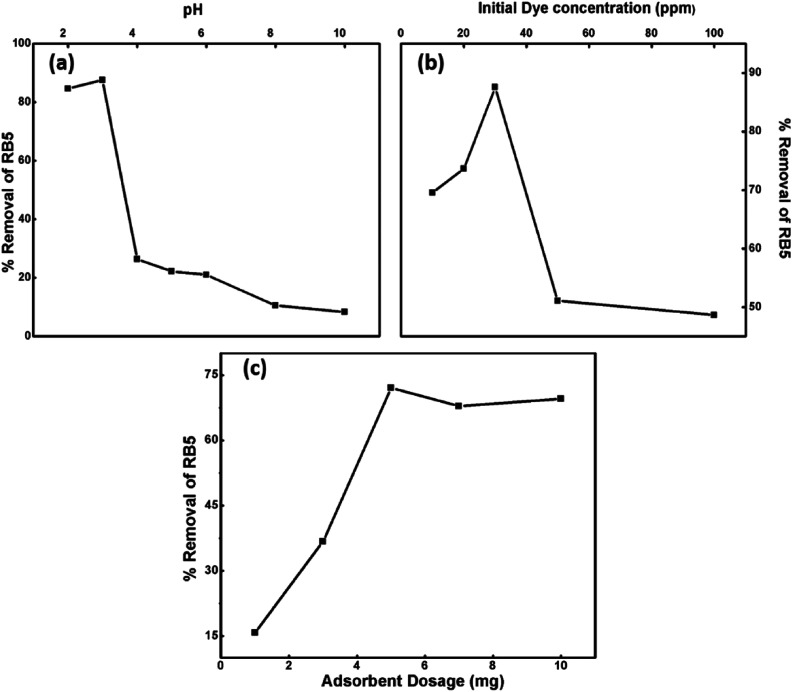
Influence of different operational parameters: (a) pH, (b) Initial dye concentration and (c) nanoparticle dosage on percentage removal of RB5.

pH is a vital parameter for contaminant removal as the degree of RB5 adsorption is initially controlled by the surface charge on Ce–Fe_3_O_4_, which is affected by the solution pH. Thus, the effects of the solution pH on RB5 adsorption were investigated in the pH range from 3 to 10 (Fig. 8a). The optimum pH for the removal of RB5 was determined *via* batch mode experiments performed on the RB5 dye with the concentration of 30 ppm and Nps dosage of 5 mg. Significant adsorption took place at acidic pH than that at alkaline pH. The maximum removal was found at pH 3. The results obtained were interpreted on the basis of zero point charge (zpc). The zpc generally lies between 6 and 6.8 mV.^45^ The effect of pH on the surface of the synthesized Nps is presented in Fig. S4,† which depicts that the zpc lies between 6 and 6.9 mV. A pH lower than the zpc generates a positive surface charge due to the accumulation of H^+^ ions or association (FeOH + H^+^ → FeOH_2_^+^) of protons. Contrarily, on moving to a higher pH, the presence of excessive OH^−^ ions or dissociation (FeOH → FeO^−^ + H^+^) makes the surface negatively charged. Being an anionic dye, RB5 is adsorbed more cogently at lower pH values. Therefore, the higher removal efficacy of the nanoadsorbent at low pH can be attributed to the electrostatic attraction between RB5 and Nps, and the reduction in removal at higher pH is linked to the electrostatic force of repulsion between these two (Fig. S5†). The maximum removal was observed for pH = 3, and therefore, this value was maintained for the remaining batch adsorption experiments.

##### 3.8.1.2. Effect of initial RB5 concentration

The impact of the initial concentration (10, 20, 30, 50 and 100 ppm) of RB5 was probed by fixing the Nps dosage amount at 5 mg and pH = 3 at ambient room temperature (Fig. 8b). The dye removal efficiency decreased with an increase in initial dye concentration. The dye removal capacity of Nps has a negative impact on the initial dye concentration. It was noticed that the removal efficiency was 87% at 30 ppm in the presence of 5 mg Nps, whereas under analogous conditions, the 100 ppm dye concentration showed 49% elimination. This effect can be explained on the basis of saturation of accessible surface active sites of the adsorbent with dye molecules. At lower concentration of dye, the number of available sites to dye ratio was optimum due to which the removal efficiency was high.

##### 3.8.1.3. Effect of nanoabsorbent dosage

The influence of Nps dosage amount on the dye removal efficiency is illustrated in Fig. 8c. Thus, dosage studies were performed to determine the capacity of the adsorbent to remove the dye. The adsorbent amounts were varied from 0.1 to 1 mg mL^−1^ against 30 ppm of RB5 at pH 3, temperature 25 °C and time of 90 min. With an increment in nanoadsorbent dose, the percentage removal of dye increased from 16 to 97% due to the greater availability of adsorbent surface sites for adsorption.

##### 3.8.1.4. Effect of contact time

To evaluate the effect of reaction time, the adsorption of RB5 in the presence of Nps with time was also analysed (Fig. 7a). Briefly, 5 mg of 3.5% Ce–Fe_3_O_4_ was added to 10 mL of 30 ppm RB5 solution. The absorbance of the solution was monitored at the wavelength of 600 nm as a function of time up to 90 min, after which saturation was observed. Fig. 7b shows the influence of time on dye removal at pH 3, and almost 97% of the RB5 has been adsorbed within 90 min. Further equilibration of the system beyond 90 min did not enhance the removal efficacy of the system. Consequently, 90 min was set as the optimum contact agitation time for the adsorption reactions.

### 3.9. Kinetic model studies

Adsorption is a physicochemical process that involves the mass transfer of solution from the liquid phase to the adsorbent surface. To study the kinetics of Nps, RB5 solution with the initial concentration 30 ppm was used under fixed adsorbent conditions. To understand the adsorption process, the pseudo-first-order model and pseudo-second-order model were analysed using eqn (3) and (4), respectively:

3 ln(*C*_o_/*C*_e_) = *Kt*

where *C*_o_ and *C*_e_ are the initial and equilibrium concentrations of dye in solution, respectively, and *K* is the rate constant (min^−1^) (eqn (3))

4 *t*/*q*_*t*_ = 1/*K*_2_*q*_e_^2^ + *t*/*q*_e_

where *q*_*t*_ and *q*_e_ denote the absorbed amount at any time and equilibrium. *K*_2_ (g mg^−1^ min^−1^) is the rate constant for the pseudo-second-order model. The best fitted results of models are shown in Fig. S6,† and the data calculated using equation (eqn (4)) is depicted in Table 3. The system follows the pseudo-second-order kinetics model with high value of correlation coefficient (*R*^2^), which may be due to chemical interaction between the nanoabsorbent and dye, thus resulting in the removal of the dye from wastewater.

**Table 3:** The fitting results of the kinetic models

First order kinetics			Second order kinetics		
*q*_e_ (mg g^−1^)	*K*_1_ (min^−1^)	*R* ^2^	*q*_e_ (mg g^−1^)	*K*_2_ × 10^−4^ (g mg^−1^ min^−1^)	*R* ^2^
69.98	0.023	0.957	370.37	1.19	0.978

### 3.10. Adsorption isotherms

The dependency of the adsorbed amount of RB5 on its equilibrium concentration in solution was investigated using the batch technique described earlier in the Experimental section, and the experimental data was analysed according to the Langmuir, Freundlich and DR isotherms to examine the type of dye molecule adsorption on the Nps surface. For wastewater treatment applications, the adsorption isotherms were analysed for evaluating the adsorption mechanism. The Langmuir model is valid for monolayer sorption on a surface with a finite number of identical sites. The distribution of dye at the solid solution interface has been described by the Langmuir equation. The well-known Langmuir model is presented by eqn (5) as follows:5
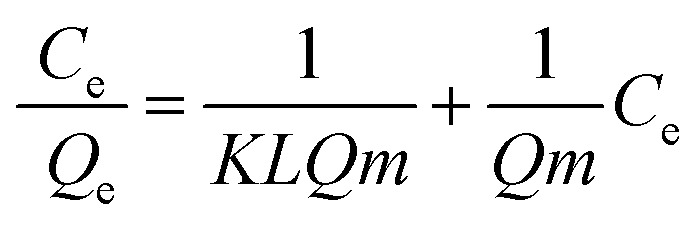

6 log *Q*_e_ = log *K*_f_ + 1/*n* log *C*_e_

where *Q*_e_ is the adsorbed amount of dye at equilibrium concentration, *C*_e_ is the equilibrium solute concentration, *Q*_m_ is the maximum adsorption capacity and *K*_L_ is the equilibrium constant for the Langmuir fit. *K*_f_ and 1/*n* are the Freundlich constants related to the adsorption capacity and adsorption intensity, respectively. Fig. S7a and b† show the fitting results of the Langmuir and Freundlich adsorption models, respectively. The calculated values are presented in Table 4. The *Q*_m_ value obtained from the Langmuir isotherm adsorption model is supported by the experimental *Q*_m_ (81.11 mg g^−1^). The *R*^2^ values of both the Langmuir and the Freundlich model indicate that the adsorption site can be occupied by only one or more RB5 molecules; this results in the formation of a monolayer/multilayer.

**Table 4:** The fitting results of different models

Isotherm	Parameters
Langmuir	*R* ^2^ = 0.988, *Q*_m_ = 84.585 mg g^−1^, *b*= 0.337 L g^−1^
Freundlich	*R* ^2^ = 0.983, *n* = 2.46, *K*_f_ = 3.227
Dubinin–Radushkevich (D–R)	*R* ^2^ = 0.726, *β* = 2.28 × 10^−7^ mol^2^ J^−2^, *Q*_s_ = 28.19 mg g^−1^

The adsorption process is mainly controlled by two methods: (a) film diffusion and (b) intraparticle (surface or pore) diffusion. The Weber–Morris equation (eqn (7)) was evaluated to determine the rate-controlling adsorption step *via* the intra-particle diffusion kinetic model.

7 *q*_*t*_ = *K*_i_*t*^0.5^ + *C*

where *K*_i_ is the intraparticle diffusion rate constant for adsorption at each step, and *C* is the intercept that symbolizes the boundary thickness.^46^ In Fig. S8b,† the graph of adsorption amount *versus* the square of time is presented, which depicts the intra-particle diffusion rate constants for each adsorption step, influencing the rate-limiting steps. The calculated values are listed in Table 4. The adsorption process can be interpreted based on the analysis of the abovementioned data: (i) the slope *K*_1_ indicates that there is an electrostatic interaction between Nps and RB5 molecules, (ii) the second step indicates that the dye molecule diffuses into the inner structure of the nanoabsorbent, which is fast diffusion, and (iii) last step represents the adsorption equilibrium process. Another well-known method to determine the type of adsorption, *i.e.* physisorption or chemisorption, is the Dubinin–Radushkevich (D–R) isotherm (Fig. S8a†) method, which is presented by the following equation:

8 log *Q*_e_ = log *Q*_s_ − *βε*^2^

where *ε* is the Polanyi potential presented by

9 *ε* = *RT* ln(1 + 1/*C*_e_)

where *Q*_s_ is the D–R constant and *β* is related to the free energy (*E*) of adsorption per molecule of adsorbate by the following equation:

10 *E* = 1/√2*β*

where *R* is the gas constant and *T* is the temperature. If the energy value is more than 8 kJ mol^−1^, it must be a chemical reaction, and if it is less than this value, it must be physical adsorption.^47^ In our studies, the energy value was determined to be 1.48 × 10^3^ J mg^−1^, revealing multilayer adsorption formation, which is based on the assumption that the adsorption sites are distributed exponentially. It also indicates that physical adsorption is the dominant phenomenon. The surface charge and hydroxyl groups are responsible for the removal of dye using the nanoabsorbent. The values of n lie between 1 and 10, which indicate the favorability of the adsorption of dye over the nanoabsorbent. Therefore, the nanoabsorbent can be considered an efficient absorbent for RB5.

### 3.11. Adsorption of a mixture of dyes onto the absorbent

**Fig. 9:**
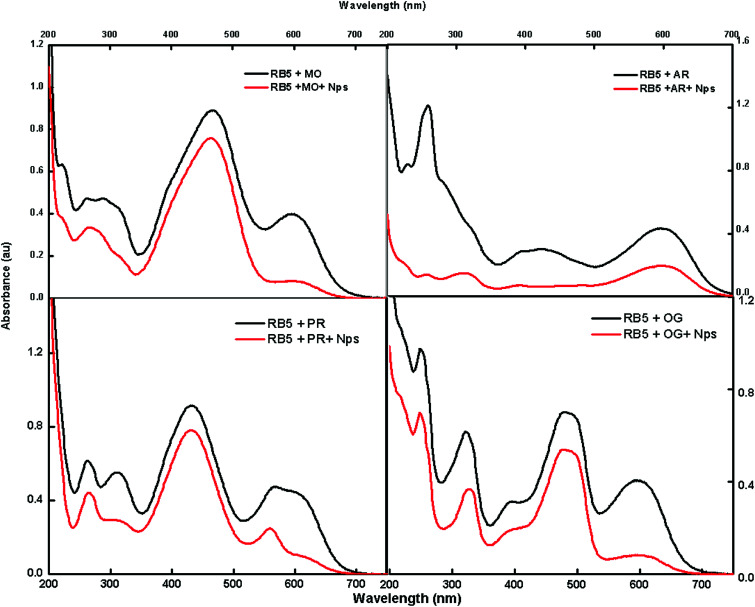
UV spectra of a mixture of dyes before and after the adsorption of dyes.

The feasibility of the absorbent towards a mixture of dyes was also tested using methylene orange (MO), Alizarin red 5 (AR), phenol red (PR) and Orange G (OG); these dyes were selected to cover the diverse range of commercially available dyes of the azo nature for the adsorption studies. Fig. 9 shows that there is a decrease in intensity after exposure to Nps.

The fast elimination capability of the nanoabsorbent further opens a new path for utilizing this type of nanomaterials for the removal of multianalytes, which can act as a good environmental remediating agent.

### 3.12. Desorption studies

The recyclability of Nps was investigated using sodium hydroxide solution as the recovery solvent. The adsorbed dye was removed by shaking in sodium hydroxide solution for 1 min. The recovered Nps were washed with water followed by ethanol. The recovered Nps were reused and the nanoabsorbent was found to be efficient after 5 cycles, *i.e.* the removal efficiency was reduced by only 5% of its initial removal (Fig. S9†). The identity of the recovered Nps was confirmed by FTIR (Fig. S10†). This study indicates the robustness of the nanoabsorbent after the treatment procedure.

### 3.13. Comparison with other adsorbents

The experiments were performed at room temperature under light. The dye concentration was 30 ppm for all the dyes employed. The pH was maintained between 3–4. A clear judgement can be made by comparing the prepared absorbent with the persisting absorbents in the literature. A comparison study was done and summarized in Table 5. It can be clearly seen that adsorption capacity (*Q*_m_; mg g^−1^) of Ce–Fe_3_O_4_ was overall greater than the previously reported magnetic systems to remove dye from wastewater.

**Table 5:** Comparison with other absorbents reported in the literature

Adsorbent	Dye	*Q*_m_ (mg g^−1^)	Reference
Fe_3_O_4_ nanosphere	Bismarck brown	33.80	48
CuO	Coomassie Brilliant blue R-250	31.75	49
	Direct Red 81	48.49	
α-Fe_2_O_3_ nanoparticles	Methyl orange	28.90	50
Sphere-like Mn_2_O_3_	Reactive black 5	14.6	51
TiO_2_ nanoparticles	Acid Red-27	38.88	52
ZnO nanoparticles	Reactive black 5	80.90	53
Ce–Fe_3_O_4_	Reactive black 5	84.58	Present work

The advantage of the present system is its adsorption capacity with magnetic retention accompanied by heterogeneous active sites, which lead to its potential application in wastewater remediation. The recyclability and easy synthesis procedure are additional advantages of this nanoabsorbent.

## 4. Conclusions

In summary, magnetite (Fe_3_O_4_) Nps with different cerium concentrations as dopants were synthesized using the hydrothermal method. The XRD analysis confirmed the presence of a single phase with an inverse final structure at all doping levels. The crystallite size was found to increase with an increase in doping concentration. The XRD results revealed the doping of Ce ions into the crystal lattice of magnetite. The saturation magnetization decreased with an increase in Ce content. The structural modification depends on the cation occupancy among the octahedral and tetrahedral sites, which influences different properties of the prepared nanoparticles. BET analysis was performed to determine the surface area of the nanoparticles, and the system with highest surface area was considered for wastewater treatment. The adsorption behavior of the 3.5% Ce–Fe_3_O_4_ was examined in detail by varying the operational parameters including pH, concentration of Nps, dye concentration and contact time. The kinetic studies and adsorption isotherms were employed to determine the mechanism of docking of the dye over the Nps surface. The removal efficiency was found to be 87%, and the efficiency was maintained even after 5 cycles. Thus, the results confirm the validity of the synthesized Nps in environmental applications *i.e.* efficacy in the removal of toxic pollutants from the environment.

## Conflicts of interest

There are no conflicts of interest to declare.

## Supplementary Material

RA-009-C9RA03252E-s001
