# Supplementary material for: Magnetically retrievable Ce-doped Fe3O4 nanoparticles as scaffolds for the removal of azo dyes
Source: RSC Adv. 2019 Jul 26;9(40):23129–41. doi: 10.1039/c9ra03252e (PMC9067296; doi:10.1039/c9ra03252e)
Supplement: RA-009-C9RA03252E-s001 [file RA-009-C9RA03252E-s001.pdf]

Electronic Supplementary Material (ESI) for RSC Advances.
This journal is © The Royal Society of Chemistry 2019

Supporting Information

Magnetically retrievable Ce-doped Fe_3O_4 nanoparticles as scaffolds for removal of azo dye

Aashima[1], Shivani Uppal[1], Arushi Arora[1], Sanjeev Gautam[2], Suman Singh[3], R.J. Choudhary[4], S.K. Mehta*[1]

1. Department of Chemistry and Centre of Advanced Studies in Chemistry, Panjab University, Chandigarh 160014, India
2. Dr S. S. Bhatnagar University Institute of Chemical Engineering and Technology (SSB UICET), Panjab University, Chandigarh, 160014, India
3. CSIR – Central Scientific Instruments Organization, Sector-30, Chandigarh 160030, India
4. UGC-DAE Consortium for Scientific Research, University Campus, Khandwa Road, Indore – 452 017, India

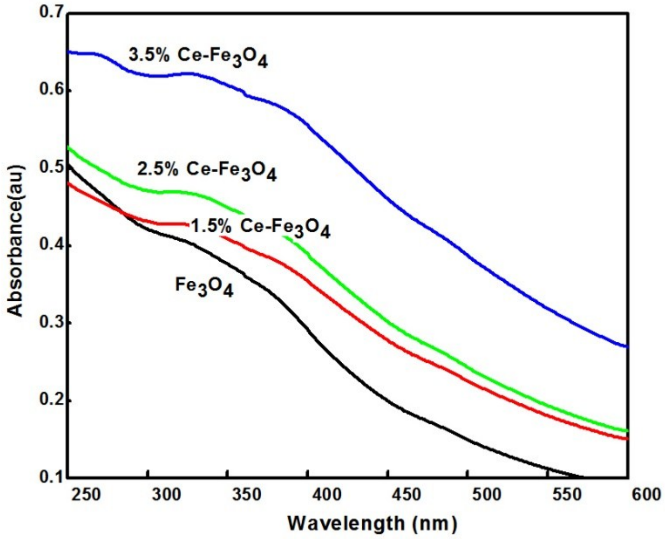

Figure S1. UV-visible spectra of synthesized samples

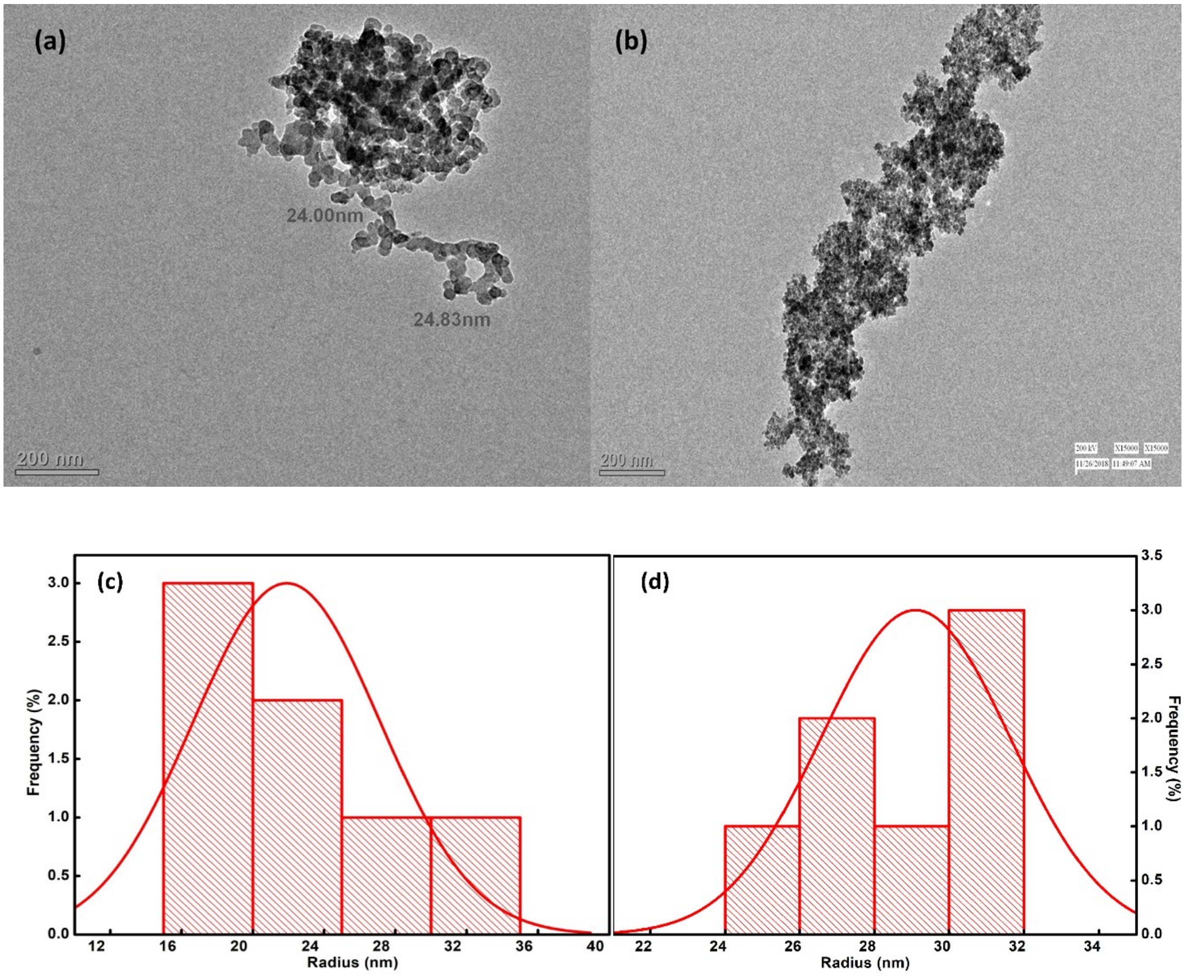

Figure S2. TEM analysis (a) Undoped (b) 3.5 % cerium doping, Histogram of (c) Undoped (d) 3.5 % cerium doping

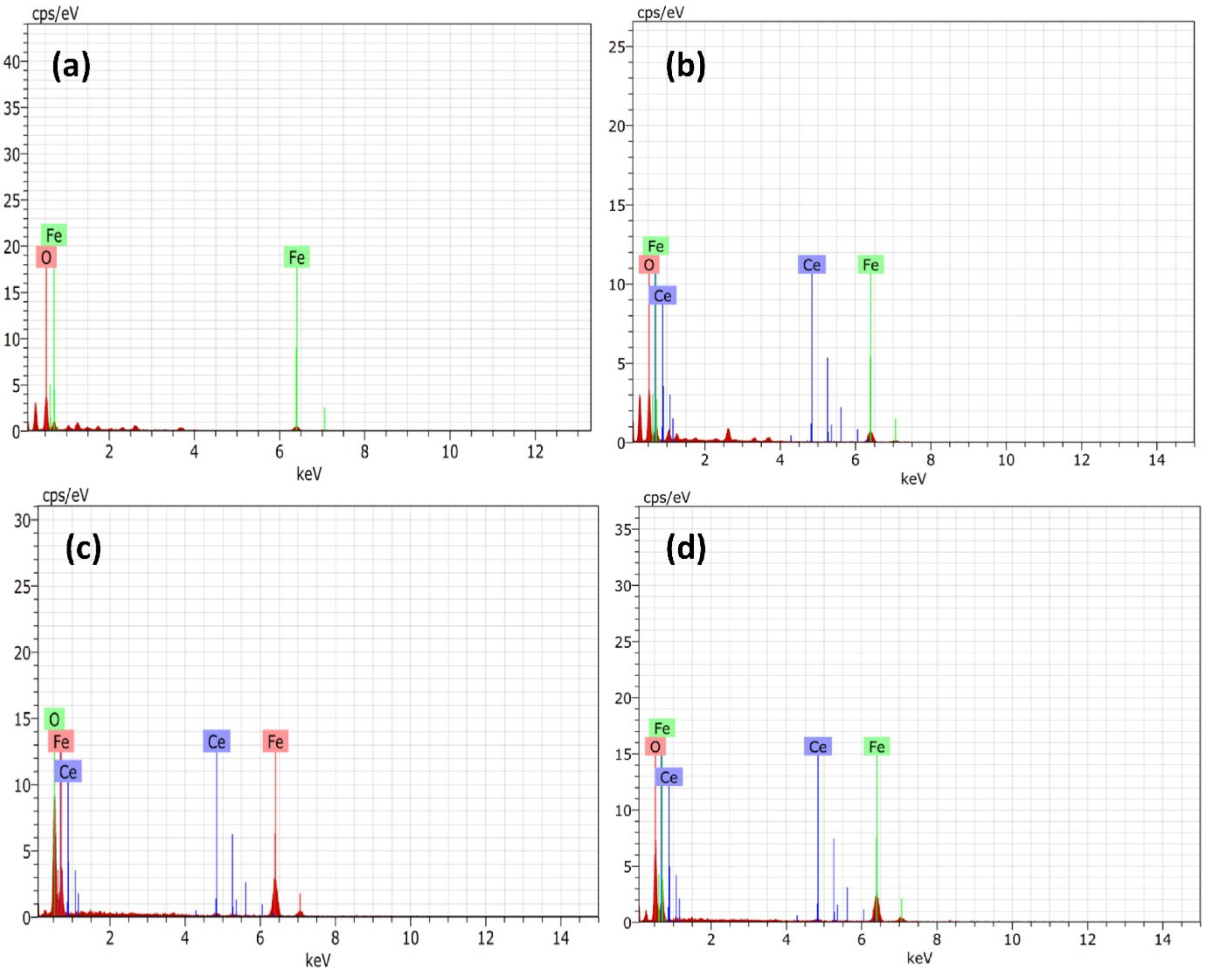

Figure S3. EDX (a) Undoped (b) 1.5 % (c) 2.5 % (d) 3.5 % cerium doping

Table S1 : Parameters calculated from BET analysis

System	Specific surface area (a) [$cm^3 g^{-1}$]	Total pore volume	Mean pore diameter [nm]	Amount of monolayer adsorption (V_m) [$cm^3 g^{-1}$]
**Fe_3O_4**	71.600	0.2976	14.491	16.450
**1.5% Ce-Fe_3O_4**	89.672	0.3249	15.039	20.603
**2.5% Ce-Fe_3O_4**	94.866	0.3567	16.625	21.796
**3.5% Ce-Fe_3O_4**	97.601	0.4611	18.898	22.424

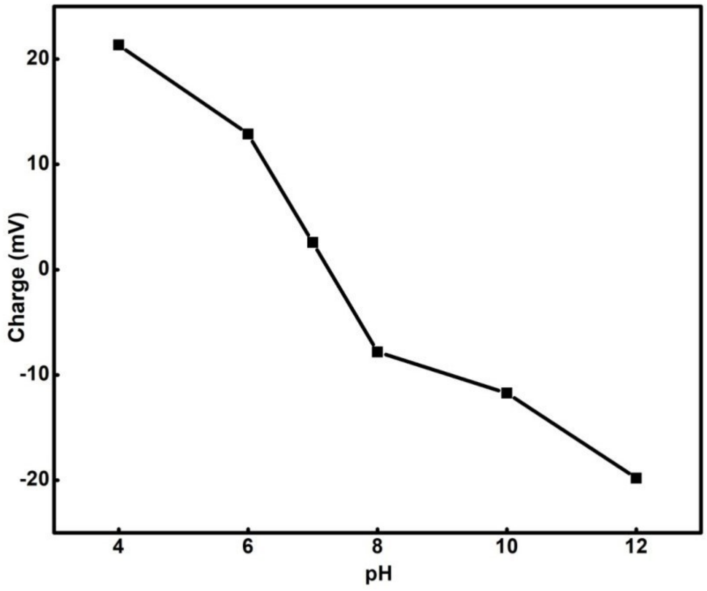

Figure S4. Variation of zeta potential charge as function of pH

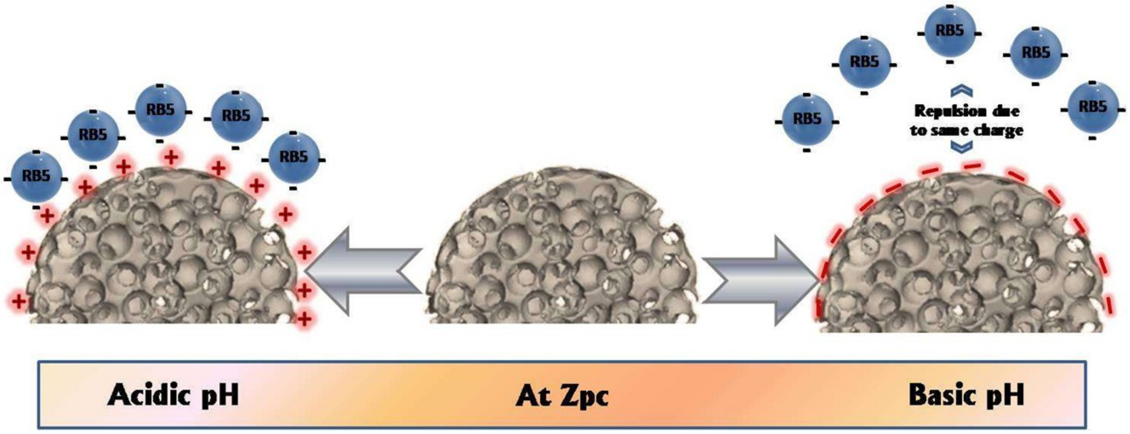

Figure S5. Role of point zero surface charge of Nanoparticles on adsorption of anionic dye RB5

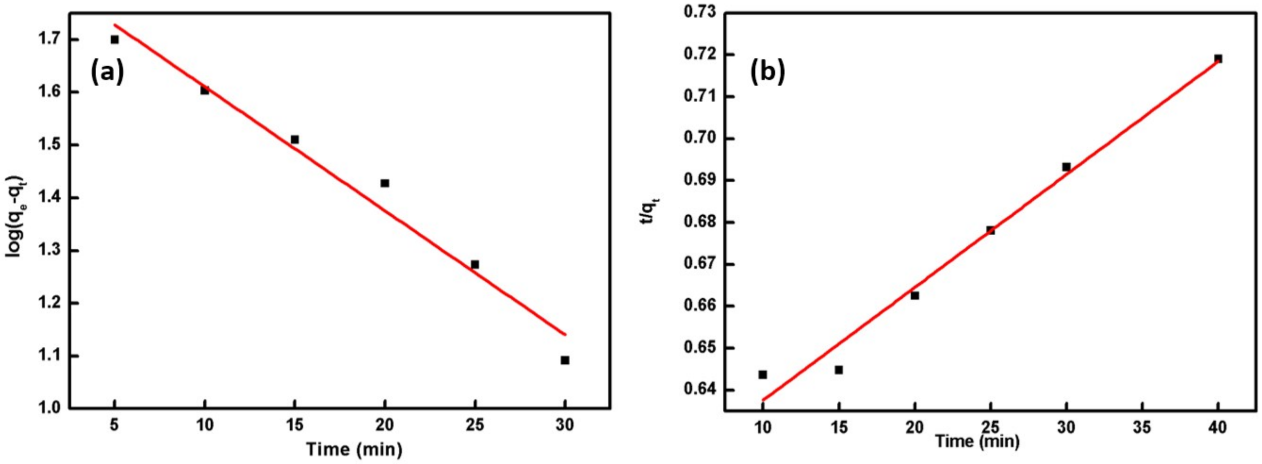

Figure S6. Kinetic models (a) Pseudo First order (b) Pseudo second order

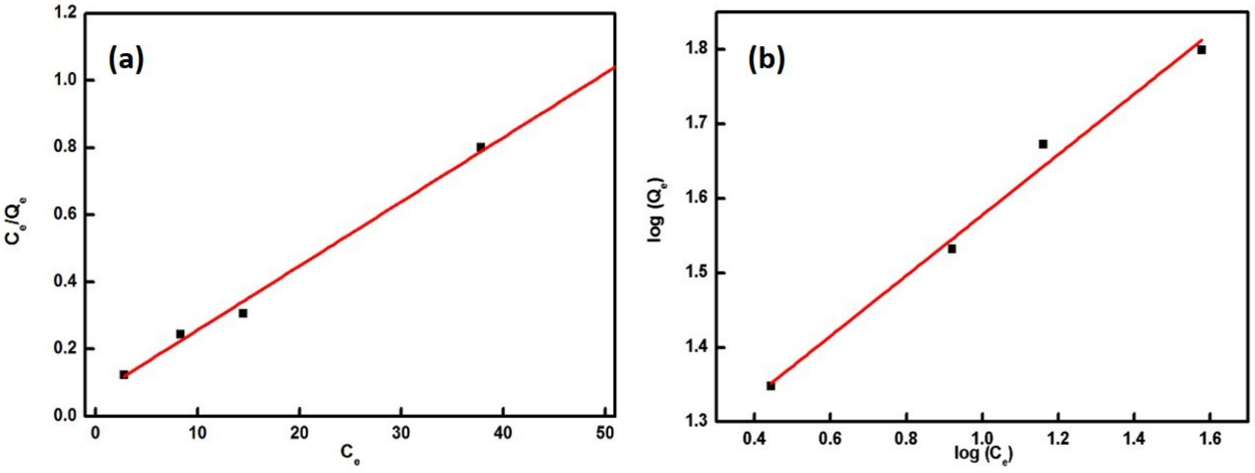

Figure S7. Adsorption isotherms of RB5 onto the Nps (a) Langmuir (b) Freundlich

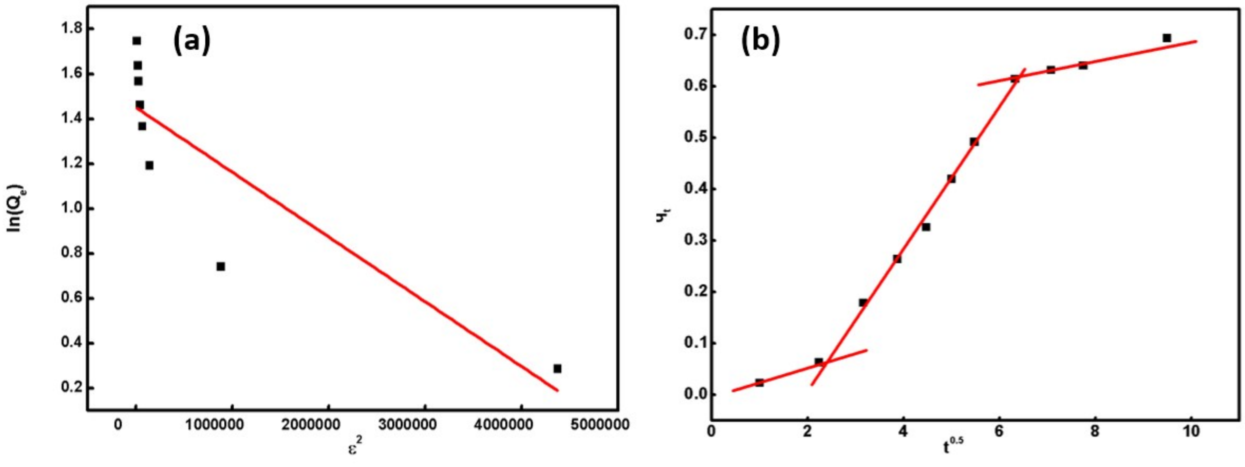

Figure S8. Adsorption isotherms of RB5 onto the Nps (a) Dubinin-Radushkevich (D-R) (b) Intra-particle diffusion models

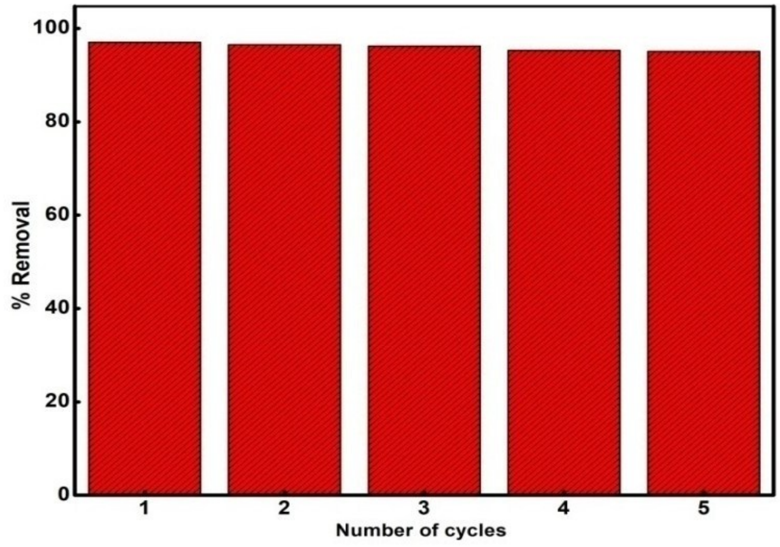

Figure S9. Recyclability of nanoabsorbent

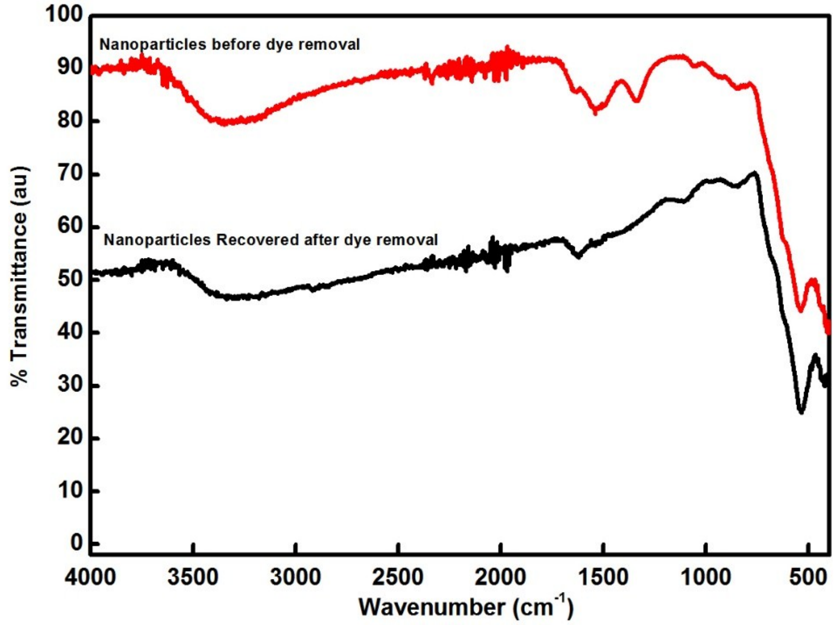

Figure S10. FTIR of recovered Nps compared with Nps before use
